# Sexting among British adults: a qualitative analysis of sexting as emotion work governed by ‘feeling rules’

**DOI:** 10.1080/13691058.2022.2080866

**Published:** 2022-06-08

**Authors:** Wendy G. Macdowall, David S Reid, Ruth Lewis, Raquel Bosó Pérez, Kirstin R. Mitchell, Karen J. Maxwell, Clarissa Smith, Feona Attwood, Jo Gibbs, Bernie Hogan, Catherine H. Mercer, Pam Sonnenberg, Chris Bonell, Natsal-4 Team

**Affiliations:** a Department of Public Health, Environments and Society, London School of Hygiene & Tropical Medicine, London, UK; b MRC/CSO Social and Public Health Sciences Unit, University of Glasgow, Glasgow, UK; c Department of Arts, Northumbria University, Newcastle upon Tyne, UK; d Independent Scholar; e Institute for Global Health, UCL London, UK; f Oxford Internet Institute, University of Oxford, Oxford, UK

**Keywords:** sexting, adults, emotion work, feeling rules

## Abstract

Sexting has generated considerable public and professional interest with concerns centring on young people, and potential harms to mental and sexual health. Little research thus far has explored the practice among adults and none has focused on the cultural norms relating to the emotional experience of sexting across different ages and genders. We conducted 40 semi-structured interviews with a diverse sample of adults aged 18-59 years in Britain on the role of digital technologies in participants’ sexual lives. In this paper, we draw on the accounts of 34 people with experience of sexting. We identified three main themes in participants’ accounts related to the emotional aspects of sexting: (1) trust, (2) desire/intimacy and (3) shame. Under each theme, we identified motivations, ‘feeling rules’, and examples of ‘emotion work’ relating to the self, the other and the dyad. We conclude that there are shared cultural norms that constitute what appropriate sexting should feel like. Interventions aiming to minimise harms arising from sexting need to build on commonly held cultural conventions regarding the ‘rules of the game’ concerning feelings as well as behaviours.

## Introduction

Sexting has generated considerable public and professional interest with concerns centring on young people, and potential harms to mental and sexual health (Ringrose et al. 2012; Cooper et al. 2016). Sexting remains variably defined (Barrense-Dias et al. 2017) but is generally understood to encompass “the sharing of personal, sexually suggestive text messages, or [self-made] nude or nearly nude photographs or videos via electronic devices” (Mori et al. 2020). Previous research has found that the term is primarily used by academics, the media and policy-makers rather than those engaging in the practice (Burkett 2015), which is corroborated by our findings presented here.

Prevalence estimates vary enormously with differences reflecting how sexting is defined, methods employed, populations and settings. Two recent meta-analyses suggest a large minority of men and women send and receive sexts, increasing with age through youth and early adulthood (Madigan et al. 2018; Mori et al. 2020), and being more common among sexual minority groups (Bauermeister et al. 2014; Currin and Hubach 2017) and those not in long-term relationships (Currin and Hubach 2017).

Among those under 18 years, sexting has been associated with sexual riskbehaviours and adverse mental health outcomes including anxiety and depression (Mori et al. 2019). Non-consensual sharing of images is associated with risk of reputational damage and poor mental health, with potentially worse outcomes for girls/women and those from religious communities (Van Ouytsel et al. 2015; Van Ouytsel et al. 2017; Anastassiou 2017; Stanley et al. 2018; Bindesbøl Holm Johansen, Pedersen and Tjørnhøj-Thomsen 2019).

As with quantitative research on sexting, most qualitative research has focused on adolescents and young adults. Such studies emphasise the importance of context, the range of activities entailed, the motivations and meanings for those involved (Ringrose et al. 2012; Walker, Sanci and Temple-Smith 2013; Burkett 2015; Anastassiou 2017; Naezer 2018), and potential benefits including: experimentation and learning (Lenhart 2009; Lippman and Campbell 2014; Yeung et al. 2014; Burkett 2015); establishing and maintaining trust and intimacy (Lenhart 2009; Lee and Crofts 2015); and pleasure and excitement (Lippman and Campbell 2014; Burkett 2015; Barrense-Dias et al. 2017; Anastassiou 2017).

Research examining sexting among adults has only recently begun to develop. Studies exploring motivations have highlighted sex-related reasons (Currin and Hubach 2019; Currin, Pascarella and Hubach 2020) and maintaining connection between partners physically apart (Currin, Pascarella and Hubach 2020). Qualitative data from an online-survey suggested excitement was the feeling most commonly associated with sexting though often accompanied by feeling anxious, naughty, good or wanted (Currin et al. 2020). Some studies have found that sexting is associated with relationship satisfaction (Parker et al. 2013), while other research links sexting with insecure attachment (McDaniel and Drouin 2015).

### Theoretical frameworks

Earlier ‘deviance discourse’ around sexting, characterised by attention to risk and harm, is being challenged by a more recent ‘normalcy discourse’, which sees consensual sexting as an everyday mode of sexual expression within intimate relationships (Döring 2014). Roberts and Ravn (2020) used Social Practice Theory in their analysis of undergraduate men’s accounts of sexting, viewing sexting as a normalised part of youth culture. Shared meanings and cultural norms centring on consent and mutuality constitute ‘appropriate’ sexting (as distinct from harassment and abuse), with a concomitant set of skills that must be learned to successfully engage in the practice (Roberts and Ravn 2020).

Amundsen (2020) drew on Hochschild’s concept of ‘emotion work’ in her analysis of interviews with adult heterosexual women, concluding that sexting constituted a form of ‘mediated intimacy work’ performed by women in which engaging with risk is used as a way to establish and enhance trust. According to Hochschild (1979), ‘feeling’ is an internal process engaged in as a precursor to external actions. Just as our actions are subject to social regulation, so are our emotions. Emotion work is the process of managing and presenting emotions in the private sphere of our lives so that they align with the social norms in particular social settings. This is in contrast to ‘emotional labour’, which involves the regulation of feelings in the public sphere and was a concept originally applied to those working in the service sector. Hochschild (1979) argued that emotions encompass: motivation (what I want to feel); feeling rules (what I think I should feel); and emotion work (what I try to feel). Emotion work involves both evocation and suppression of feelings and can involve managing one’s own and others’ emotions. Hochschild (1979) further identified three emotion-management strategies in undertaking emotion work: cognitive (trying to modify thoughts in the hope of changing the feelings associated with them); bodily (using the body to create a desired emotion); and expressive (attempting to change expressive gestures to change inner feelings).

By applying this theoretical framework to our analysis of the accounts of sexting from a diverse sample of adults, we aimed to understand not only the emotional motivations for sexting (as Amundsen did) but also how individuals gauge what they should and should not feel, and how they work to manage their own emotions and those of others while engaging in the practice. Our analysis focused on the core elements of Hochschild’s framework rather than her concepts of exploitation, ideology and class conflict given our focus was on intra- and inter-personal processes rather than broader structures or discourses.

The research was undertaken as part of exploratory qualitative research to understand topics for inclusion in the fourth British National Survey of Sexual Attitudes and Lifestyles (Natsal-4).

## Methods

We conducted 40 semi-structured interviews with adults resident in Britain (England n=25,Scotland n=10, and Wales n=5) in May-June 2019. Half the interviews, conducted by London School of Hygiene and Tropical Medicine (LSHTM) researchers, focused primarily on the role of digital technologies in participants’ sexual lives and secondarily on sexual wellbeing. In the other half, conducted by University of Glasgow researchers, the focus was reversed. Participants were recruited by a research-recruitment agency, using quota-sampling to ensure variation in terms of age, gender, ethnicity, relationship status, sexual identity and area of residence/area deprivation (Table 1).

Six researchers conducted the interviews face-to-face in venues of participants’ choosing, generally their homes or university offices. In all but one interview, only the interviewer and participant were present; one participant requested a friend be present. Prior to seeking consent, participants were provided with study information and given the opportunity to ask questions.

Interviewers used a guide with questions about the participant’s experience of sexual/intimate relationships, and use of digital media generally and in relation to sex. This included: sending or receiving sexual text messages, pictures or videos, or having sexual encounters using audio or video. Interviewers probed for technologies used; motivations; the meaning and feelings associated with such activities; and perceived benefits and harms. Interviews were audio-recorded and transcribed verbatim.

Transcripts and field-notes were entered into NVivo 11. An inductive thematic analysis was first undertaken, before deductively applying an analytic framework informed by Hochschild’s emotion-management perspective (Hochschild 1979). This was prompted by the prominence in participants’ accounts of how sexting should feel and their efforts in trying to bring their own emotional experiences in line with this. A coding framework was developed with codes covering motivations, feeling rules and emotion work, which was then augmented and refined through coding participant accounts. Axial coding then identified cross-cutting themes. Pseudonyms have been assigned to participants to protect confidentiality.

In terms of reflexivity, the research team was diverse in gender, age and sexual identity. Discussions around the time of data analysis established that all conceived of sexting as a ‘normal’ practice imbued with meaning and shared a desire to move away from a risk-based approach, to one that acknowledged the potential for a range of outcomes including positive ones.

Ethical approval for the study was granted by LSHTM’s research ethics committee (reference 17046 26/4/2019).

## Findings

In their accounts of using digital media in a ‘sexual way’, participants distinguished between text and picture messages and, as reported elsewhere (e.g. Burkett 2015), rarely used the term sexting, instead referring to ‘sexy, ‘dirty’, ‘racy’ or ‘rude’ text messages and ‘images’, ‘pictures’, ‘nudes’ and ‘dick pics’.

Among the 40 participants, 34 had sent or received a sext (text or image). Of these 34: all had received text-based sexts and 29 had sent them; 28 had received image-based sexts and 24 had sent them; 16 identified as female and 18 as male; five were aged 18 to 20 years old, 27 were aged 20 to 49, and two were aged 50 or over; six were from Black, Asian or other minority ethnic communities; and ten identified as lesbian, gay or bisexual (Table 1). The six participants with no direct experience of sexting described their attitudes towards it or friends’ experiences.

We identified three cross-cutting themes centring on the emotional aspects of sexting: (1) trust, (2) desire/intimacy and (3) shame. Aligning with Hochschild’s framework, under these themes, we explored motivations, feeling rules and emotion work (Table 2).

### Trust

Sexting was described as ‘a very trusting thing to do’ in the context of participants describing how they could make themselves vulnerable to shaming or onward sharing through engaging in the practice. In terms of motivation, the sharing of intimate images was seen as a way that trust could be evoked, demonstrated and reinforced with an associated set of feeling rules that ‘I should feel trust’, ‘they should feel they can trust me’, and ‘we should have an interaction which feels mutually trusting’. That mutuality was a key rule is illustrated by the quote from Josy’s account below. In being sent images, she recognised the trust that had been placed in her, which she wanted to honour and expected to be reciprocated.

I just think they’re private, and they’ve sent it to you in trust…So, I don’t want to betray that trust because it is an intimate picture… I wouldn’t want them to do it to me, so I wouldn’t do that, that to them.

(Josy, Female, 30s, White British, married, lesbian)

While the rules of trust principally centred on fears of images ‘getting out’, not all participants were overly worried about this possibility (‘it’s just a body’). This was a view expressed more commonly by men irrespective of sexual identity. Many women spoke about protecting against the harms associated with onward sharing by not showing their faces in pictures (‘at the end of the day if your face isn’t in it you can say it wasn’t me’). As Amundsen (2020) identified, the inclusion of identifying characteristics was seen as higher risk, which therefore demanded and demonstrated higher levels of trust. It is likely that the norms concerning trust are more salient for women than men because of the unequal gendered consequences of onward image-sharing (Ringrose et al. 2013). Among the gay men interviewed, there appeared to be a culture of onward image-sharing. Oliver described how he had shared pictures that had been sent to him and that he ‘expected’ others might have done likewise with images of himself. . Work to suppress negative emotions by avoiding thinking about onward sharing allowed him to feel all right about this.

Interviewer: Has anyone ever shared images of you with other people without your permission?

Not to my knowledge but they could have quite easily, I expect they have… I’ve shared images with lots of people so I’d imagine that they have been shared.

Interviewer: How does that make you feel?

Sometimes just, like, ignorance is bliss I suppose!

(Oliver, Male 20s, White British, in relationship, gay)

Some accounts highlighted the receipt of unsolicited sexts as an exception to the rules around trust. For example, Riya felt justified in onward sharing as a legitimate response to having received unsolicited images since, in sending these unsolicited, the sender had breached a norm of reciprocity.

This kid called [name]… he was just sending us pictures and I was going, ‘if you don’t stop sending us these pictures’ I went ‘I’m going to send them on’. He sent us another one so I sent it to where he worked, to one of the main lads on his lunch break. So it literally went round all his mates. And like, he had nothing to talk about.

Like, like literally I was going, ‘I’d feel embarrassed. I’ve seen like, I’ve seen bigger party sausages, mate’, like literally.

(Riya, Female 30s, Asian British, heterosexual)

Participants commonly described the emotional work involved in managing their feelings of trust and vulnerability when sexting, particularly in the early stages of a relationship while trust was being built. One of Hochschild’s emotion-management strategies (cognitive work) is illustrated in Angela’s account below. The cognitive work involved suppressing conflicting emotions within herself to align with the rule that you should trust your partner.

Interviewer: When you shared sexual images, that idea of being exposed potentially, was that in your mind?

It was in my mind… me thinking that I’m going to get exposed. Just that I was thinking that I’m doing this but some people get exposed for this so I’d have to think whether I can trust the person and if I’m doing the right thing. So yes, I just preferred not to do it that way. But after a while, I got used to it. Like we got that trust and I knew that he wouldn’t do anything…

(Angela, Female <20, Black African, single, heterosexual)

When the rules of trust were breached, participants worked to manage their conflicting feelings regarding blame. This tension is illustrated in Zoe’s account of when her friend had a sext shared in school. Zoe was managing her own conflicting feelings arising from her evaluation of her friend’s behaviour and also trying to direct her friend’s emotional response to the fallout from this incident, within a gendered, victim-blaming discursive context.

I felt awful and at the same time I was, like, ‘you stupid girl’. But I didn’t want her to think that, like, that wasn’t okay. I wanted her to know that people, girls didn’t feel any different about her. Some girls would be horrible, but I wanted her to, like, feel, like, ‘look, at the end of the day, he’s a dickhead for sending them’. It wasn’t her fault.

(Zoe, Female, 20s, White British, cohabiting, heterosexual)

### Desire/intimacy

Most participants spoke about demonstrating or reinforcing desire/intimacy as their primary motivations for sexting. These motivations were linked to the role of sexting as a form of personal intimacy that was either a precursor to a potential future sexual encounter; a form of foreplay evoking feelings of sexual desire/arousal; a sexual practice in its own right; or a way of reinforcing intimacy after sex. There was an implied set of feeling rules governing the management of sexual desire during sexting. As with trust, for sexting to feel enjoyable mutuality was key, as illustrated in the quote from Richard below.

Interviewer: What were your motivations…?

Just to make time for the two of you to say you want to arouse them. To think OK, at some point you want to have sex and you’re thinking about them. You want them to be desired as well, same as yourself… Or even if you’ve had sex to even send a message the next day about that. Because, as I say, it’s not, it’s not such a regular occurrence anymore…

Interviewer: So why would you send it the next day, what are your motivations?

Just because you feel closer again. It’s very easy to lose yourself a little bit… you’ve both got your own battles going on with the kids and with your own life and work and so on that when you do have sex and you feel that intimacy again, you think ‘why don’t we do it more often?’ It almost makes you feel like you haven’t got the kids, you’re back to as you were.

(Richard, Male, 40s, White British, married, heterosexual)

As well as being mutually enjoyable, participants’ accounts highlighted rules that sexting should feel flirtatious and fun rather than overly sexual, intimidating, ‘sordid’, routine or one-sided, further illustrating the feeling rule that sexting should involve mutual desire. Angela, while reflecting on the differences between sharing text- and image-based messages, described the emotion work involved in building and evoking sexual feelings in herself and her partner through sexting. For her, moving from text descriptions to the exchange of images was a natural progression and a way to ‘make real’ mental images evoked through words. It was important for her that her own feelings of desire and arousal were shared and understood by her partner. Also important for her was that the images looked ‘appealing’ and portrayed her well. This ‘impression management’ appeared to be as much about how the images made her feel about herself (i.e. desirable) as how they would make her partner feel about her (i.e. desired).

Yes. So, to me, I’d say sexting is more like descriptive rather than visual. So, like, you’re describing sexual parts of the body that you’re most fond of or things you would like to do with your partner or somebody. And, like, it’s more descriptive in a sense, like you’re going in more detail so that you can get the other person to understand and visualise mentally like what you’re feeling, like what emotions you’re feeling… Your initial reaction is to want to send images. Because you’re envisioning what you’re thinking in your mind and trying to bring it to life. But sending images, I’d say, is just like nudes, so sending areas of the body. But I feel like it has to look appealing, like not just send it. Like when people just send images, I feel like it’s about how you portray yourself within the images as well.

(Angela, Female, <20, Black African, single, heterosexual)

As a type of foreplay, people often spoke of how sexting could help build sexual tension/anticipation and many spoke about ‘teasing’ their partner. James enjoyed the sense of control resulting from sexting with his partner:

I do it, and then he asks for more and I’m like ‘yeah, that’s enough’… Yeah, because I just like, I like the control… I have what he wants. If I don’t give it, it winds him up.

Interviewer: And how does that make you feel?

Quite, it makes you feel powerful.

(James, Male, 20s, White British, gay]

In contrast to the earlier finding that a key feeling rule was that sexting should be mutually enjoyable, some participants (mainly women and gay men) expressed a willingness to engage in the practice, despite feeling less keen on it than their partner. In such instances, sexting was performed for the sake of the recipient, prioritising the recipient’s emotional experience over their own within a context of gendered norms and sexual scripts prioritising male pleasure. Hannah reported no benefits to herself (apart from a fleeting good feeling from being complimented). In the face of a disconnect with a feeling rule (‘it should be mutually enjoyable’), this left her feeling ‘uncomfortable’ and ‘weird’, emotions that then required management.

I just wanted to keep him happy… more than anything, I just… And even though I didn’t feel comfortable I did it because… they liked it so…

Interviewer: Are there any benefits in doing that? To you?

No. Sometimes they, they, they give compliments and you do feel good for about five minutes and then you… I felt kind of weird then.

(Hannah, Female, 20s, White British, cohabiting, heterosexual)

In another example, Oliver, a young gay man, talked about the cultural expectations of image-sharing. As above, there is a disconnect with the rule that sexting should be a product of mutual desire.

Interviewer: Why did you send those pictures?

Generally, because they would ask for them… I was never actually that interested in receiving pictures, didn’t really sort of do it for me… A lot of people are on there so it’s like, it’s expected basically… I was always interested in seeing, you know, face pictures…

Interviewer: Why’s that?

Because that was more important, how someone’s face was, as opposed to, you know, their dick or their body etc., so I would prefer to see a face. So, I would often ask for face pics… But [it] was almost like a bit sort of lame in a way to, to ask for that…

(Oliver, Male, White British, 20s, in a relationship, gay)

The notion of desire raises the questions of what it is that is desired and what work is done to evoke desire. Hannah related a mismatch in expectations between men and women about what was desirable and arousing. To her, pictures of penises were not attractive, though she implied that the intention of boys sending pictures of their penises was to impress her (and not to arouse her). She evoked desire in partners and a feeling of being desirable through creating and sending erotic but partly clothed images, though she went on to suggest that men cannot do similarly.

Boys always send pictures of their penises because they think it’s impressive. But you would never want to send a picture of your body parts because I just don’t think they’re attractive and I don’t think penises are attractive either…

Interviewer: So, you were keeping it more kind of subtle and a bit covered up and then turns him on but…

Yeah, whereas boys can’t really do that because what’s attractive about a pair of boxers? Nothing. Whereas underwear [female] is attractive.

(Hannah, Female, 20s, White British, cohabiting, heterosexual)

In sexting, the sender renders themselves vulnerable. In his account of his experiences of sexting, Simon talked about how he had been made to feel undesired. With the rule of being desired breached, he described how he needed to manage his emotions in order to feel good about himself.

Oh, sometimes it is, you might send a picture or something and they’re really insulting and you think… ‘that’s so fucking personal’. But what can you do? You just. Next. Got to feel good. You know?

(Simon, Male, 30s, White British, single, gay)

Some participants, particularly those in long-term and/or long-distance relationships, referred to feeling rules for sexting in terms of the importance of maintaining intimacy and emotional connection with their partner. In this way, sexting may be regarded as work done in maintaining intimacy in relationships. In her account Alana, described emotion work to evoke a sense of emotional and sexual connection with a partner.

Interviewer: What were your motivations in sending those pictures… to each other?

Obviously to keep the relationship interesting. And we’d go a long time without seeing each other. So, it was just that, to keep that bond, some connection between us.

(Alana, Female, 20s, White British, single, heterosexual)

In another example, Richard described how text-based sexting with his wife is not just the sex’, it is aiming to strengthen the relationship and evoke feelings of intimacy and mutual value.

You want to feel valued in a relationship and I’m sure your partner wants to feel valued.

Interviewer: So, it’s a way of you feeling valued as well, she, that interaction by text.

Yeah, so it’s not just the sex, it’s about being close again.

Interviewer: How have those interactions made you feel?

Good. Valued… I think it strengthens your relationship because it’s very easy to become distant and drift apart.

(Richard, Male, 40s, White British, married, heterosexual)

### Shame

While not a motivation, some participants’ accounts suggested ambivalence and shame about sexting, which they needed to manage. In terms of feeling rules, this ambivalence appeared to be rooted in whether their sexting practices aligned with their wider beliefs about culturally appropriate expressions of sexuality. Simon, reflecting on sharing text-based sexts, described feelings of concern, embarrassment and regret, suggesting a feeling rule that one should feel ashamed of expressing one’s sexuality in this way. He describes the cognitive emotion work involved in managing his feelings so that he could feel comfortable with sexting.

At the time I’m always, like, I’m single, it’s consensual… it fits you know. So I don’t ever have any regret… I’m not doing anyone any harm, so it’s good. I guess, analysing it, because I’m, analysing the next day if I was reading back and thinking ‘God, I wouldn’t have sent that if… I had been sober… if I’d been in another frame of mind. And then I’ll think ‘God, what if anyone else saw that?’ Another time, that’s obviously the sexual side of your nature… When you’re not in that zone at all, you read that and you think… ‘oh for fuck sake, did I actually send?’ You always feel embarrassed… At the time, you’re all, the fact that you’re so in tune, so confident to… to explore all these things… that is the spur on… So, when you think ‘what are they going to say?’ And it’s like, and it’s well received… And it’s like ‘yeah, I feel empowered’ that I’m able to say that and then the next day you’re thinking delete, delete! So, a bit of both.

(Simon, Male 30s, White British, single, gay)

Ambivalence about sexting is further illustrated Eva’s account. She found it both ‘exciting’ and ‘disgusting’ at the same time and seemed confused by her conflicting emotions which she needed to manage. As with Simon’s account above, there seems to be internal conflict regarding the ‘appropriateness’ of expressing her sexuality in this way.

So how does it make you feel..?

So, it’s exciting at the, yeah, it’s exciting. But then sometimes I’ll be like, ‘it’s actually disgusting and I don’t know why I’m doing it’. And then sometimes I’ll be like, ‘I don’t even want to see you’. I don’t know, it’s weird, it’s different emotions.

(Eva, Female 20s, white, living alone, bisexual)

## Discussion

This is the first qualitative study of sexting among a diverse sample of adults that has systematically explored the various emotional aspects of sexting. In contrast, previous studies have more narrowly explored motivations (Currin et al. 2020; Currin and Hubach 2019) and feelings (Currin et al. 2020) but have not explored feeling rules and the full breadth of emotional work involved. Using Hochschild’s concept of emotion work as an analytic framework, we explored emotional motivations for sexting, feeling rules and emotional work that participants undertook to align their feelings with these rules.

In our sample, the primary motivations for engaging in sexting related to demonstrating and/or reinforcing sexual desire and maintaining intimacy. This finding resonates with other research on adult sexting that has found sex-related motivations to be key (Currin and Hubach 2019). However, while previous research has suggested that the primary motivation is to initiate sex, participants also spoke of its role in reinforcing feelings of closeness and connection after sex. While sex-related reasons have been identified in research among young people, they tend to be listed among a wider set of motivations for engaging in the practice (Burkett 2015; Bianchi et al. 2017).

Our findings also resonate closely with those of others in terms of the centrality of trust (Amundsen 2020). In order for sexting to feel all right, it needed to align with the rules about trust. Evaluating and managing feelings of trust, especially while it is still being built, involved considerable emotion management, particularly for the women in our sample. Like Ringrose et al. (2013), we found evidence of the greater risks women perceived, reflecting the highly gendered social consequences of onward image-sharing. In her analysis of the accounts of the sexting practices of heterosexual women, Amundson (2020) concluded that sexting involves a constant negotiation of risk and trust but, rather than simply attempting to avoid risk, women harness this as a resource with which to establish and enhance trust, a conclusion echoed in our findings.

There was evidence in our sample that the salience of the rules of trust appeared to vary by social location. For example, among men, while mutual feelings of trust were idealised, they appeared less critical for some, perhaps because the consequences of onward image-sharing were seen as less detrimental for men (Anastassiou 2017). Furthermore, the gay men in our sample spoke about a culture of more widespread onward image-sharing. The importance of body presentation among men who have sex with men has been highlighted in previous research (Silberstein et al. 1989) and the use of nudity specifically in online presentations has been found not just to be driven by sex-related reasons but involve gratifications involving empowerment or affirmation from others (Lemke and Merz 2017).

Mutuality was also central. There were, however, important areas of ambivalence and inequality. Some, mainly female, participants engaged in sexting for the primary benefit of their partners with the associated emotional work of prioritising partners’ emotional experiences over their own and of deriving emotional pay-off from making their partners feel good. This suggests a normative context of the prioritisation of male pleasure being influential on sexual scripts.

While there has been little research on sexting among adults, there is some evidence of positive outcomes such as increased relationship satisfaction (Parker et al. 2013). This has, however, as noted above, been challenged by research with married couples which has suggested that sexting is associated with relationship insecurity and dissatisfaction (McDaniel and Drouin 2015). It was evident in some of our accounts that when individuals’ experiences of sexting aligned with their understanding of feeling rules (and there were mutual feelings of trust and sexual desire), then it could foster positive feelings of closeness and connection, especially within long-term or long-distance relationships. As such this ‘online intimacy’ appeared to enrich existing ‘offline’ relationships (Lomanowska and Guitton 2016). While previous research has highlighted the role of women as ‘intimacy workers’ (Elliott and Umberson 2008), many men in our sample also placed value on such work and spoke of engaging in sexting to engender or maintain feelings of intimacy.

It was also evident in these accounts that sexting intersects with other social practices and the cultural conventions governing appropriate ways of behaving and feeling. It was striking that in several accounts, men and women expressed ambivalence about their (consensual) sexting practice. Ambivalence appeared to be rooted in whether their sexting aligned with beliefs about socially acceptable ways to experience and express sexuality, such participants undertaking emotional work to validate such practices.

### Limitations

One limitation of our study derived from the fact that participants were not purposively selected on whether they had experience of sexting so not everyone we interviewed could discuss the subject from personal experience. Our sample was diverse in terms of sexual orientation and included adults of various ages, allowing us to explore the emotions associated with sexting across a variety of behaviours and groups. Although it included men and women, all were cisgender. Another limitation is that we did not purposely frame and focus questions in terms of emotion work and feeling rules, only using this analytic framework at the analysis stage. Finally, our analysis focused on intra- and inter-personal processes and did not attempt to examine systematically the broader structural influences on, and social discourses surrounding these.

## Conclusion

Findings from this study offer useful evidence to inform interventions and further research on sexting as a normal, and potentially positive, part of intimate relationships. In terms of further qualitative research there is more to be said about the nuances of the ‘rules’ within specific age, gender, sexuality and ethnic sub-groups. Existing legal and public health responses to sexting generally focus on adolescents and emphasise risks rather than balancing risks and pleasures, and the skills required to do so (Cooper et al. 2016). Interventions to promote sexual wellbeing need to build on commonly held cultural conventions regarding the ‘rules of the game’ and attend to both behavioural and feeling rules. This has the potential to inform more pragmatic, nuanced and acceptable interventions.

## Figures and Tables

**Table 1:** Characteristics of participants

		Sent *or* received a sext (text or images) (n)	Not sent or received a sext (n)	Sent *and* received a sext (text and images) (n)
**Total**		**34**	**6**	23
**Gender**	Female	16	4	9
	Male	18	2	14
**Age**	<20	5	0	4
	20s	16	1	11
	30s	10	0	7
	40s	1	3	0
	50+	2	2	1
**Ethnicity**	White British	28	5	19
	Asian/British/Pakistani/Indian	3	1	1
	Black/British/African	2	0	2
	Mixed ethnicity	1	0	1
**Relationship Status**	Single	17	1	12
	In a relationship	17	5	11
**Sexual Identity**	Heterosexual	24	6	16
	Bisexual	4	0	2
	Lesbian	2	0	1
	Gay	4	0	4

**Table 2:** Feeling rules in the accounts of sexting practice

Theme	Feeling Rules	Practice to evoke and suppress emotion	Examples from participant accounts
**Trust**	I should feel trust, a partner should feel trust, we should feel mutual trust. I should feel respectful of trust given. I should feel badly about betraying trust. I should feel more comfortable and disinhibited and that sexting is appropriate when trust is present.	Evocation of feelings of trust. Suppression of feelings of doubt.	Sharing personal text and images as a way of building, demonstrating and maintaining trust. Reinforcing existing trust through continued sexting and sharing more intimate or personally identifiable pictures. Perceptions of comfort when trust is felt. When trust not present seen as foolish, inappropriate or inviting trouble.
**Desire**	I should feel desired and desire my partner, we should feel mutual desire. Sexting should feel enjoyable, flirtatious and fun and not overly sexual, intimidating or routine.	Evocation and maintenance of feelings of interest, desire in partner.	Creating and sharing erotic text and images as a way of evoking sexual interest, arousal and excitement in self and other. Experiencing arousal, desire and enjoyment from sexts received. Experiencing sexual enjoyment and pleasure from sexualised interaction when physically distanced. Keeping tone light and humorous rather than heavily sexual.
	I should not feel neutral about sexting partner. I should not feel uninterested or distant from partner I should not feel bored with sexual life. Feelings of rejection should not be present. I should not feel intimidated or harassed by unwanted sexual contact.	Suppression of sexual ambivalence, habituation and rejection.	Encouraging self to send sexual images or to interact sexually in novel or arousing ways though text. Revisiting and appreciating positive sexual experiences. Waiting for partners to sext to avoid rejection. Blocking, ignoring or challenging unwanted sexual contact. Seeking support from others by sharing unwanted or uncomfortable experiences.
**Shame**	Sexting should feel right. I should feel comfortable with the interaction. I should feel that sexting is appropriate in context. I should not feel ashamed of sexting within ‘the rules’.	Evoking good feelings of comfort, confidence and fun.	Pushing self to engage, demonstrate confidence or seek positive reinforcement. Justifying in context such as when single, at the beginning of relationships, when separated from each other, or in long term relationships.
	Sexting should not feel negative, pressured, awkward or confrontational. I should feel embarrassed, ashamed if engaged in inappropriate sexting such as non-consensual or overly sexual.	Suppressing negative feelings of shame and discomfort or evoking them in others.	Suppressing negative feelings, moving on to another partner.
